# Paraneoplastic lipase and amylase production in a patient with small-cell lung cancer: case report

**DOI:** 10.1186/s12885-016-2167-7

**Published:** 2016-02-17

**Authors:** Andrea Casadei Gardini, Marita Mariotti, Alessandro Lucchesi, Sara Pini, Martina Valgiusti, Sara Bravaccini, Angelo Del Monte, Marco Angelo Burgio, Giorgia Marisi, Dino Amadori, Giovanni Luca Frassineti

**Affiliations:** Department of Medical Oncology, Istituto Scientifico Romagnolo per lo Studio e la Cura dei Tumori (IRST) IRCCS, Meldola, Italy; Biosciences Laboratory, Istituto Scientifico Romagnolo per lo Studio e la Cura dei Tumori (IRST) IRCCS, Meldola, Italy

**Keywords:** Small-cell lung cancer, Paraneoplastic syndrome, Serum lipase, Pancreatic amylase, Response marker

## Abstract

**Background:**

Small-cell lung cancer (SCLC) is known to express antigens of both the neural crest and epithelium, and to secrete polypeptide hormones and enzymes. Anecdotal reports correlate lung cancer with marked hyperamylasemia, and a review of the literature reveals only one case of metastatic SCLC linked to high paraneoplastic lipase production.

**Case presentation:**

We present the case of a patient with metastatic SCLC who showed both lipase and pancreatic isoamylase elevation in the absence of acute pancreatitis.

Chemotherapy resulted in a rapid reduction in serum lipase and in pancreatic isoamylase which was correlated with the radiological response of the tumor to therapy. Lipase and pancreatic isoamylase expression in tumor cells from the lung biopsy was confirmed by immunohistochemical staining.

**Conclusions:**

This is a very rare case of paraneoplastic syndrome linked to metastatic SCLC. The enzymes secreted could be used as markers of response to treatment until clonal selection mechanisms and intratumor heterogeneity induce changes in biochemical characteristics and consequently in tumor behavior.

## Background

Small-cell lung cancer (SCLC) is known to express antigens of both the neural crest and epithelium, and to secrete polypeptide hormones and enzymes. The most common paraneoplastic endocrine manifestations in SCLC are the syndrome of inappropriate antidiuretic hormone secretion (SIADH) and Cushing’s syndrome [1, 2]. Anecdotal reports correlate lung cancer with marked hyperamylasemia, and a review of the literature shows only one case of metastatic SCLC linked to high paraneoplastic lipase production [3].

We present the case of a patient with metastatic SCLC who showed both lipase and pancreatic isoamylase elevation in the absence of acute pancreatitis. Chemotherapy induced a rapid decrease in serum lipase and in pancreatic isoamylase which was correlated with radiological confirmation of response of the tumor to therapy.

## Case presentation

**Fig. 1:**
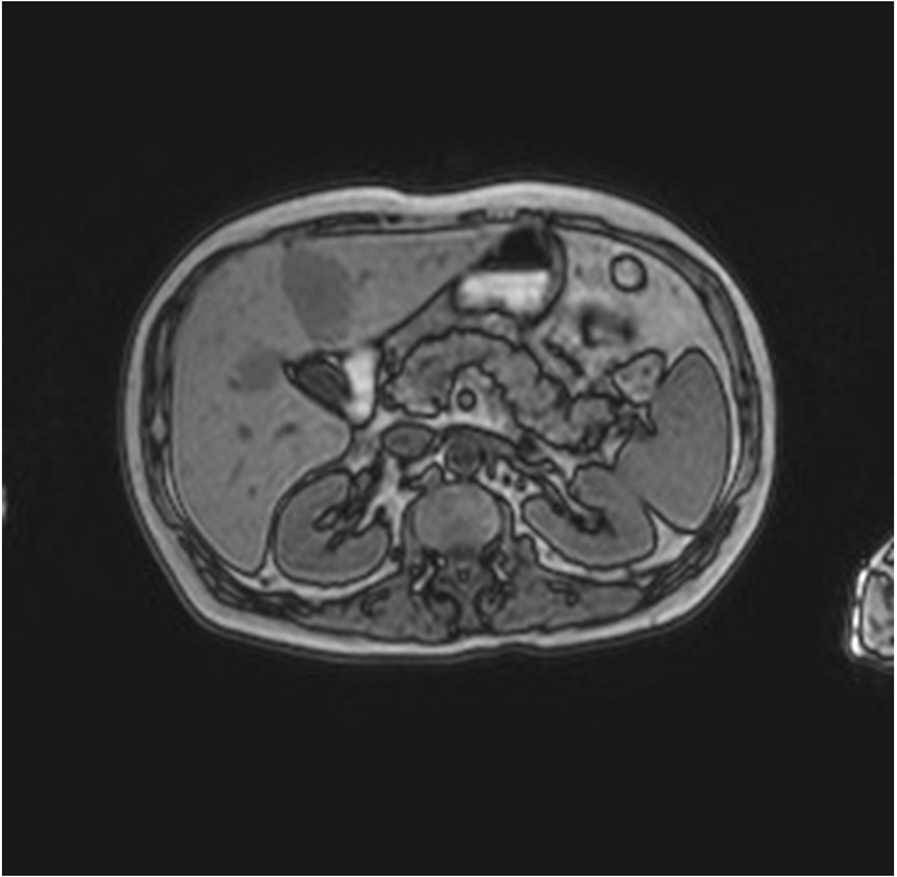
MRI was negative for acute pancreatitis and other diseases

A 54-year-old man presented with small cell lung cancer (SCLC) and metastases of the brain, liver, adrenal glands and mediastinal lymph nodes. Immunohistochemically, tumor cells were positive for CD56 and TTF1. The patient had a 30-year history of cigarette smoking and his past medical history was significant for arterial hypertension and diabetes. He only took medications for back pain and did not drink alcohol. Given his performance status, we considered first-line chemotherapy with cisplatin and etoposide. A blood test before the start of chemotherapy showed normal renal and liver functions but high serum lipase levels (1343 U/L, normal value 13–60 U/L). Further tests revealed elevated levels of neuron-specific enolase (NSE) (173.9 μg/L), carcinoembryonic antigen (220.7; normal value < 5 μg/L) and serum amylase (379; normal value 1–100 U/L). Although progastrin-releasing peptide was recently identified as a biomarker of SCLC, we chose not to analyze it as it has not yet been introduced into clinical practice. Pancreatic isoamylase value was 339 U/L (normal value 17–115 U/L). Serum calcium and other electrolytes were normal. The patient was asymptomatic. Magnetic resonance imaging (MRI) of the abdomen performed to exclude pancreatic lesions and/or pancreatitis was negative (Fig. 1).

In the absence of clinical and radiological findings of pancreatitis, chemotherapy was begun with cisplatin and etoposide. After the first cycle of chemotherapy, serum lipase values decreased sharply to 592 U/L and NSE levels fell to 24 μg/L. At the end of the third cycle, a further reduction in serum lipase (270 U/L), pancreatic isoamylase (128 U/L) and NSE levels (21.6 μg/L) was observed and was correlated with the radiological response of the tumor to therapy. The restaging CT scan showed a significant reduction in the number and size of both primary and metastatic lesions.

**Fig. 2:**
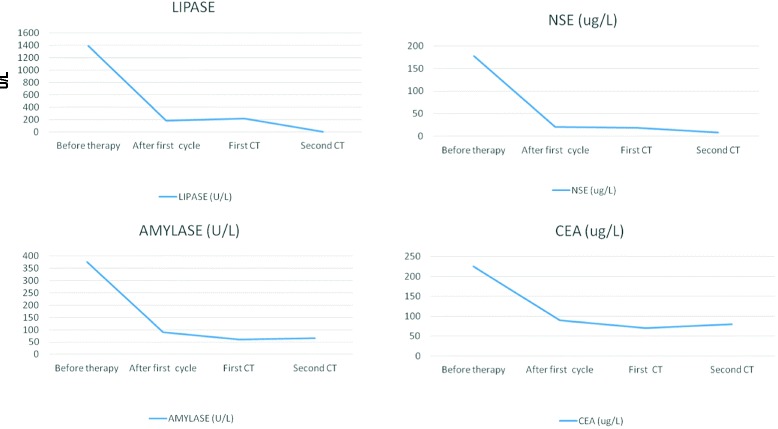
Marker trends

Given the patient’s positive response to treatment and his good performance status, we decided to continue chemotherapy, obtaining a normalization of the serum lipase concentration (12 U/L vs. initial baseline level of 1343 U/L) after the sixth cycle. In addition, total serum amylase dropped to 74 U/L, NSE to 15.7 μg/L and CEA levels to 20.1 μg/L (Fig. 2). A second restaging CT scan of the chest and abdomen showed a further reduction in the size of the target lesions. However, a brain MRI revealed disease progression with multiple lesions and panencephalic brain radiotherapy was started. One month after the end of chemotherapy the patient was hospitalized due to the onset of epileptic seizures and progressive physical decline. Palliative care was begun but the patient died 1 week after admission (7 months after diagnosis).

## Conclusion

SCLC is believed to arise from Kulchitsky cells which originate in the neural crest and are present beneath the epithelial lining of the lungs. It is also reported to have antigenic markers of both the neural crest and epithelium and to secrete polypeptide hormones and enzymes [1]. Cushing’s disease, which is related to ectopic adrenocorticotropic hormone secretion, and the syndrome of inappropriate antidiuretic hormone secretion are the most frequent paraneoplastic endocrine complications in SCLC. There is anecdotal evidence of a correlation between lung cancer and marked hyperamylasemia. In fact, amylase-producing tumors of the lung include adenocarcinoma and undifferentiated small-cell carcinoma [2].

A review of the literature revealed only one case of metastatic SCLC with elevated paraneoplastic lipase production which was associated with SIADH, a second paraneoplastic condition [3, 4]. Our patient showed both lipase and pancreatic isoamylase elevation in the absence of acute pancreatitis or other non cancer-related disorders.

Pancreatic amylase and lipase activity have been found in some human non-pancreatic organs such as the salivary glands, stomach, duodenum, large pancreatic ducts, extra-hepatic bile ducts and gallbladder. Elevation of amylase and lipase is seen in nonmalignant disorders such as acute pancreatitis, trauma, pancreatic abscess, cholecystitis, common bile duct obstruction, cholangitis and perforation or intestinal obstruction. A rare case of nonmalignant pancreatic hyperenzymemia was also reported by Gullo [4]. The syndrome, named after the researcher (Gullo’s syndrome), is characterized by a fluctuating increase in pancreatic enzyme serum levels in the absence of pancreatic diseases.

In pathologic tissue, immunoreactivity for one or more pancreatic enzymes is found in the epithelial cells of salivary gland pleomorphic adenoma, squamous cell carcinoma of the esophagus, adenocarcinoma of pancreatic and biliary origin, and colorectal and gastric adenoma and adenocarcinoma. Terada et al. suggested that some tumors continue to express these enzymes after neoplastic transformation [5, 6]. Isolated reports of hyperamylasemia in lung cancer would appear to be due to an increase in a salivary-like amylase isoenzyme [4].

**Fig. 3:**
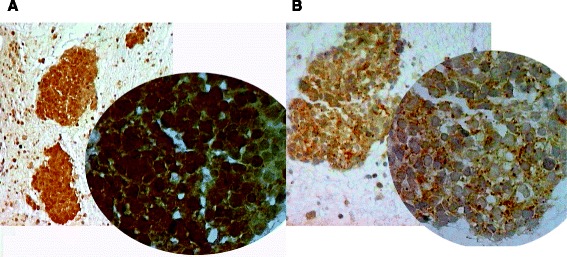
Histological sections of the primary tumor showing 90 % (intensity 1+/2+) (**a**) and 100 % (intensity 3+) (**b**) amylase and lipase immunopositivity

As far as we know, this is the first report of a case of elevated lipase production associated with increased pancreatic isoamylase expression. There is strong evidence that both hyperlipasemia and pancreatic-type hyperamylasemia represent a paraneoplastic phenomenon. In our patient, diagnostic tests and the absence of clinical symptoms excluded inflammatory conditions or pancreatic disorders. The rapid decrease in enzyme levels shortly after the start of chemotherapy appeared to be closely related to the reduction in size and number of primary and metastatic lesions. Lipase and pancreatic isoamylase expression was confirmed by immunohistochemistry and the percentage of immunopositive tumor cells was evaluated with respect to the total number of tumor cells of the biopsy. Staining intensity was scored from 0 to 3+. In particular, histological sections of the primary tumor showed 90 % (intensity 1+/2+) (Fig. 3a) and 100 % (intensity 3+) (Fig. 3b) immunopositivity for amylase and lipase, respectively. Both enzymes showed marker-like behavior as their expression paralleled the response of the primary tumor to chemotherapy.

In conclusion, this is a very rare case of paraneoplastic syndrome linked to metastatic SCLC. The enzymes secreted could be considered for use as markers of response to treatment until clonal selection mechanisms and intratumor heterogeneity induce changes in biochemical characteristics and consequently in tumor behavior.

## Consent

Written informed consent for publication of their clinical details and clinical images was obtained from the patient relative of the patient. A copy of the consent form is available for review by the Editor of this journal.

## Abbreviations

SCLC: small-cell lung cancer
SIADH: syndrome of inappropriate antidiuretic hormone secretion
